# Liver-Derived TGF-β Maintains the Eomes^hi^Tbet^lo^ Phenotype of Liver Resident Natural Killer Cells

**DOI:** 10.3389/fimmu.2019.01502

**Published:** 2019-07-03

**Authors:** Cathal Harmon, Gráinne Jameson, Dalal Almuaili, Diarmaid D. Houlihan, Emir Hoti, Justin Geoghegan, Mark W. Robinson, Cliona O'Farrelly

**Affiliations:** ^1^School of Biochemistry and Immunology, Trinity Biomedical Sciences Institute, Trinity College Dublin, Dublin, Ireland; ^2^School of Medicine, Trinity College Dublin, Dublin, Ireland; ^3^Liver Unit, St. Vincent's University Hospital, Dublin, Ireland; ^4^Department of Biology, Maynooth University, Maynooth, Ireland

**Keywords:** TGF-β1, liver-resident NK cell, TBET, microenviroment, Eomes

## Abstract

The adult human liver hosts a complex repertoire of liver resident and transient natural killer (NK) cell populations with diverse phenotypes and functions. Liver resident NK cells are CD56^bright^ NK cells defined by a unique expression profile of transcription factors and cell surface markers (Eomes^hi^Tbet^lo^TIGIT^+^CD69^+^CXCR6^+^CD49e^−^). Despite extensive characterization of the phenotype of liver resident NK cells, it remains unclear how factors within the liver microenvironment induce and maintain this unique phenotype. In this study, we have explored the factors regulating the phenotype of liver resident NK cells. Isolation of healthy liver resident NK cells from donor liver perfusate and *in vitro* culture results in the gradual loss of the characteristic Tbet^lo^ phenotype, with the cells increasing Tbet expression significantly at day 7. This phenotypic loss could be halted through the dose-dependent addition of liver conditioned media (LCM), generated from the *ex vivo* culture of liver biopsies from healthy organ donors. TGF-β, but not IL-10, replicated the Tbet suppressive effects of LCM in both liver resident and peripheral blood NK cells. Furthermore, blocking TGF-β receptor signaling using the inhibitor SB431542, reversed the effect of LCM treatment on liver resident NK cells, causing the loss of tissue resident Eomes^hi^ Tbet^lo^ phenotype. Our findings identify liver-derived TGF-β as an important component of the liver microenvironment, which acts to regulate and maintain the phenotype of liver resident NK cells.

## Introduction

Large populations of tissue resident natural killer (NK) cells have been identified in a diverse range of tissues including liver, lung, lymph node, bone marrow, and uterus (1). However, the tissue-specific factors regulating resident NK cell phenotype and function remain largely unknown. The adult human liver hosts a diverse repertoire of liver resident and transient NK cell populations with differing phenotypes and functions, whose roles in liver homeostasis and disease remain poorly defined.

Liver resident NK cells are enriched in the CD56^bright^ NK cell subpopulation and are defined by their unique expression of the transcription factors Eomes and Tbet, and a range of cell surface markers including TIGIT, CD69, CXCR6, and the absence of CD49e (Eomes^hi^Tbet^lo^Hobit^+^TIGIT^+^CD69^+^CXCR6^+^CD49e^−^) (2– 6). This liver resident phenotype is absent in peripheral blood NK cell populations. Expression of the chemokine receptor CXCR6 is thought to be particularly important for the retention of resident populations within the liver due to constitutive expression of the ligand (CXCL16) in the liver sinusoid.

Despite extensive characterization of the phenotype of liver NK cells, it remains unclear how factors within the liver microenvironment contribute to the induction and maintenance of this unique phenotype. Our group has previously described the wide range of cytokines and growth factors present in healthy adult liver which can maintain and propagate NK cells (7–10). However, this work did not explore specific liver resident NK cell phenotypes. Previous research identified that liver resident NK cells produce significantly less IFN-γ compared with corresponding peripheral blood NK cell populations, and liver conditioned media (LCM) was sufficient to suppress IFN-γ in peripheral blood CD56^bright^ cells (2, 3), suggesting that liver-derived soluble factors are capable of regulating NK cell populations.

In this study we demonstrate that LCM can maintain the liver resident NK cell phenotype *ex vivo*, as well as suppress Tbet and Eomes expression in peripheral NK cells (which mirrors the Tbet phenotype observed in liver resident NK cells). Furthermore, we show TGF-β, produced in the liver microenvironment, suppresses Tbet and Eomes expression in liver resident NK cells and peripheral blood NK cells. Understanding how the liver microenvironment induces and maintains liver specific immune cell populations may identify novel therapeutic targets capable of regulating local immunity and tissue repair.

## Materials and Methods

### Collection of Liver Perfusate During Orthotopic Liver Transplantation (OLT)

Samples were collected from donor livers (*n* = 10) during orthotopic liver transplantation at St. Vincent's University Hospital. During retrieval, the donor aorta and superior mesenteric vein were flushed with University of Wisconsin (UW) solution (Bristol-Myers Squibb, Uxbridge, UK) at the time of exsanguination. The liver was flushed again with UW solution after excision of the organ until all blood was removed and the perfusate appeared clear, at which time the liver was placed in a container with UW solution and packed on ice for transportation. Donor livers were transplanted within 12 h. At implantation, after completion of the upper inferior cava anastomosis, livers were flushed with normal saline through the portal vein to wash out the UW before reperfusion. This wash-out fluid was collected from the inferior vena cava; the UW transportation solution was also collected. A matched donor blood sample was taken at the time of organ retrieval. Peripheral blood was obtained from anonymised blood donors from the Irish Blood transfusion Board (IBTS). All protocols were approved by St. Vincent's University Hospital Ethics Committee and the Trinity College Dublin School of Medicine Research Ethics Committee, in accordance with the ethical guidelines of the 1975 Declaration of Helsinki.

### Isolation of Hepatic Mononuclear Cells From Liver Perfusate and Peripheral Blood

Hepatic mononuclear cells (HMNCs) were isolated from donor liver perfusates, as previously described (11), by filtration through 70 μm filters (BD Biosciences, Erembodegem, Belgium) followed by centrifugation at 1,200 rpm for 10 min. The supernatant was aspirated and the cells resuspended in RPMI 1640 medium, supplemented with 10% fetal calf serum and 1% penicillin/streptomycin (Gibco, Wicklow, Ireland). HMNCs were separated from this suspension by density gradient centrifugation using Ficoll-Paque™ PLUS (GE Healthcare, Uppsala, Sweden) and residual red blood cells were removed by adding red cell lysis solution (Sigma, Wicklow, Ireland). Matched peripheral blood mononuclear cells (PBMCs) were also isolated by density centrifugation.

### Preparation of Liver Conditioned Media

Wedge biopsies, taken at the time of transplantation, were used to generate tissue conditioned media. Tissue samples were measured and weighed. Tissue was then cut into sections measuring approximately 0.5 cm × 0.5 cm × 0.5 cm. These were placed in a 24-well-culture plate and 500 μl of X-VIVO (Lonza Biologics, Slough, UK) media was added to each well and incubated for 72 h at 37°C. Following incubation, the supernatant was centrifuged to remove cell debris and stored at −20°C until use.

### Isolation of NK Cells From Liver Perfusate and Peripheral Blood

CD3^−^CD56^+^ NK cells were isolated from fresh liver perfusates and healthy donor peripheral blood by negative selection using NK cell isolation kits (130-092-657; Miltenyi Biotech, Teterow, Germany, and 480054; BioLegend, CA, USA), as per the manufacturers protocol. Briefly, mononuclear cells were labeled with a biotin conjugated antibody cocktail against lineage specific targets. Anti-biotin microbeads were then added, and the NK cells were separated using a magnetic cell sorting (MACS) LS column. Cells were then cultured in RPMI 1640 supplemented with 10% fetal calf serum and 1% penicillin/streptomycin. In addition, NK cells were sorted into two populations CD56^bright^ CD16^+/−^ and CD56^dim^ CD16^++^ using a FACS Aria cell sorter (BD Biosciences).

### Culture of Hepatic and Peripheral Blood NK Cells

Isolated NK cells were plated at 5 × 10^5^/ml in round-bottomed 96 well-plates in RPMI 1640 supplemented with 10% Human AB serum and rhIL-15 (2 ng/ml) for 7 days in the presence or absence of liver conditioned media (5/10% v/v), TGF-β (5 ng/ml), or IL-10 (10 ng/ml). Media was changed every 2–3 days.

### Phenotypic Analysis of NK Cells

HMNCs, PBMCs and cultured NK cells were stained with fluorescently labeled monoclonal antibodies to determine the phenotypic differences between hepatic and peripheral NK cells. The following antibodies were used: CD45 (HI30) BV510, CD3 (UCHT1) BV421 or Pacific Blue, CD56 (NCAM16.2) BV650 or APC, CD16 (3G8) PE-Cy7, CXCR6 PE-CF594. Intracellular staining was performed using FoxP3 staining buffer (00-5523-00, eBiosciences, San Diego, CA, USA) and the following antibodies were used: Eomes (WD1928) AF488 and T-bet (ebio4B10) PE (BioLegend). Dead cell exclusion was carried out using fixable viability stain 780 (BD Biosciences). Flow cytometric analysis was carried out using an LSR Fortessa (BD Biosciences) or a FACS Canto II (BD Biosciences) and data was analyzed using FlowJo (Version 7.6.5, Tree Star, Ashland, OR, USA).

### Statistical Analysis

Statistical analysis was carried out using Prism GraphPad Version 5.0. For comparison of two unmatched groups, Mann Whitney *U*-test was used. Comparison of more than two unmatched groups was performed using Kruskal-Wallis test, with Dunn's multiple comparison test. For paired comparisons, Friedman test with Dunns multiple comparison tests was used for more than two groups, Wilcoxon signed rank test was used for comparison of two matched groups. Within an experiment, ^*^p < 0.05, ^**^p < 0.01, and ^***^p < 0.001, respectively.

## Results

### Liver Resident NK Cells Are Characterized by a Eomes^hi^Tbet^lo^CXCR6^+^ Phenotype, Which Is Gradually Lost Upon *ex vivo* Culture

**Figure 1:**
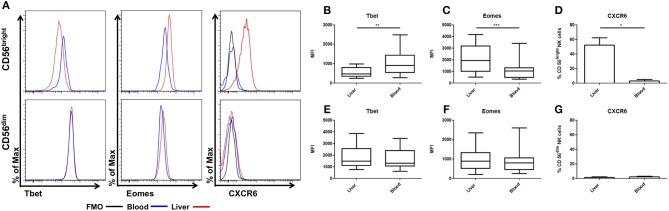
CD56^bright^ NK cells have unique tissue resident phenotype. Mononuclear cells isolated from liver perfusate (LP) and matched peripheral blood (PB) were stained with monoclonal antibodies. NK cells were identified from the CD45^+^ lymphocyte gate as CD56^+^CD3^−^. CD56^bright^CD16^−/+^ and CD56^dim^CD16^+^ subsets were then gated and non-viable cells were excluded by FVS780 staining. **(A)** Representative histograms of Tbet, Eomes and CXCR6 expression in LP and PB NK cell subsets. **(B)** The MFI of Tbet in CD56^bright^ NK cells. **(C)** The MFI of Eomes in CD56^bright^ NK cells. **(D)** Percentage of CXCR6 positive CD56^bright^ NK cells. **(E)** The MFI of Tbet in CD56^dim^ NK cells. **(F)** The MFI of Eomes in CD56^dim^ NK cells. **(G)** Percentage of CXCR6 positive CD56^dim^ NK cells. Data presented as mean ± SEM **(D,G)** or box and whisker plots with minimum and maximum values **(B,C,E,F)**. Data was analyzed using Wilcoxon matched pairs test (*n* = 10; *p < 0.05, **p < 0.01, and ***p < 0.001).

NK cells from donor liver perfusate and matched peripheral blood were analyzed for expression of Eomes, Tbet, and CXCR6. As previously described, CD56^bright^ NK cells from liver perfusate displayed reduced expression of Tbet (LP: MFI 554 ± 69.6, PB: MFI 1074 ± 189.9, *p* = 0.0024, Figures 1A,B). Liver CD56^bright^ NK cells had increased expression of Eomes compared to matched blood cells (LP: MFI 2092 ± 337, PB: MFI 1126 ± 248, *p* = 0.0005, Figure 1C). Liver CD56^bright^ cells also express CXCR6, which is almost undetectable on peripheral blood counterparts (LP: 52.1 ± 10.1%, PB: 3.1 ± 1.9%, *p* = 0.0223, Figure 1D). In contrast, CD56^dim^ NK cells from liver perfusate resemble their peripheral blood counterparts with no significant differences in Eomes, Tbet, or CXCR6 (Figures 1E–G).

In order to determine whether the phenotype of hepatic NK cells is permanently altered by residing within the liver microenvironment, NK cells were isolated from liver perfusate and cultured in RPMI supplemented with human AB serum, to replicate the peripheral blood microenvironment. It was necessary to supplement this culture with IL-15 (2 ng/ml) in order to maintain NK cell viability and expression of CD56 (12, 13). NK cells were cultured for 7 days with the culture media replaced every 2–3 days.

**Figure 2:**
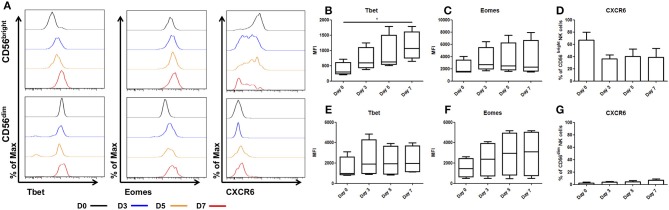
Tbet expression increases in liver resident NK cells cultured *ex vivo*. NK cells isolated from liver perfusate (LP) were cultured for 7 days and stained with monoclonal antibodies to assess transcription factor expression. **(A)** Representative histograms of Tbet, Eomes and CXCR6 expression from cells at day 0 (black line), day 3 (blue line), day 5 (orange line), and day 7 (red line). **(B)** MFI of Tbet in CD56^bright^ NK cells from LP at day 0, 3, 5, and 7. **(C)** MFI of Eomes CD56^bright^ NK cells from LP at day 0, 3, 5, and 7. **(D)** Percentage of CXCR6 positive CD56^bright^ NK cells from LP at day 0, 3, 5, and 7. **(E)** MFI of Tbet in CD56^dim^ NK cells from LP at day 0, 3, 5, and 7. **(F)** MFI of Eomes in CD56^dim^ NK cells from LP at day 0, 3, 5, and 7. **(G)** Percentage of CXCR6 positive CD56^dim^ NK cells from LP at day 0, 3, 5, and 7. Data presented as mean ± SEM **(D,G)** or box and whisker plots with minimum and maximum values **(B,C,E,F)**. Data was analyzed using Friedman test, with Dunn's multiple comparison test (*n* = 5; *p < 0.05).

Upon *ex vivo* culture the unique phenotype of CD56^bright^ hepatic NK cells is lost, with Tbet increasing significantly between day 0 (MFI 461.5 ± 255.5) and day 7 (MFI 1214 ± 572, *p* = 0.042, Figures 2A,B). Eomes expression increased but this did not reach statistical significance (day 0 MFI 2147 ± 632 and day 7 MFI 3497 ± 1500, *p* = 0.3, Figure 2C). Expression of the chemokine receptor, CXCR6, showed a trend toward decreasing on hepatic CD56^bright^ NK cells upon *ex vivo* culture, however this did not reach statistical significance (day 0, 71.8 ± 10.2%, and day 7, 40.3 ± 10.4%; *p* = 0.052, Figure 2D).

In CD56^dim^ hepatic NK cells, Tbet remained stable during culture with the MFI ranging between MFI 2007 at day 0 and MFI 1550 at day 7 (*p* = 0.69, Figure 2E). Eomes expression increased between day 0 (MFI 1463 ± 516) and day 7 (MFI 3364 ± 1807, Figure 2F) but this did not reach statistical significance. CXCR6 expression in CD56^dim^ hepatic NK cells increased marginally, however this increase was not statistically significant and even at day 7 only represents a small minority of the total CD56^dim^ population (Day 0 2.5 ± 1.3% vs. day 7 6.6 ± 2.4%; *p* = 0.3, Figure 2G).

### The Eomes^hi^ Tbet^lo^ Phenotype of Liver Resident NK Cells Can be Maintained *ex vivo* by Supplementing With Liver Conditioned Media

**Figure 3:**
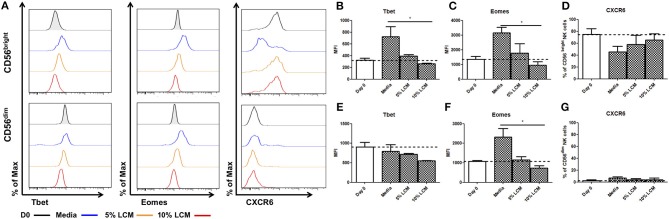
Liver resident phenotype can be maintained by the addition of liver conditioned media. NK cells isolated from liver perfusate (LP) were cultured for 7 days, supplemented with 5 or 10% v/v liver conditioned media (LCM) and stained with monoclonal antibodies to assess transcription factor expression. **(A)** Representative histograms of Tbet, Eomes and CXCR6 expression from cells at day 0 (black line), day 7 untreated (blue line), day 7 5% LCM (orange line), and day 7 10% LCM (red line). **(B)** MFI of Tbet CD56^bright^ NK cells from LP at day 0 and 7 with LCM treatments. **(C)** MFI of Eomes in CD56^bright^ NK cells from LP at day 0 and 7 with LCM treatments. **(D)** Percentage of CXCR6 positive CD56^bright^ NK cells from LP at day 0 and 7 with LCM treatments. **(E)** MFI of Tbet in CD56^dim^ NK cells from LP at day 0 and 7 with LCM treatments. **(F)** MFI of Eomes in CD56^dim^ NK cells from LP at day 0 and 7 with LCM treatments. **(G)** Percentage of CXCR6 positive CD56^dim^ NK cells from LP at day 0 and 7 with LCM treatments. Data presented as mean ± SEM. Data was analyzed using Friedman test, with Dunn's multiple comparison test (*n* = 5; *p < 0.05).

Hepatic CD56^bright^ NK cells have a unique pattern of Eomes and Tbet expression, which is lost upon long term culture. In order to determine the effect of the liver microenvironment on these transcription factors, we assessed Eomes and Tbet expression after *ex vivo* culture supplemented with LCM (*n* = 5). LCM significantly reduced the expression of Tbet in liver resident NK cells, in a dose dependent manner, to a level similar to that seen at day 0. At day 7, the MFI of Tbet in untreated CD56^bright^ NK cells was 724.3 ± 170.4 MFI, rising from 323.7 ± 34.3 MFI at day 0. This was reduced to 391.0 ± 29.8 MFI with 5% LCM and further reduced to 261.7 ± 11.9 with 10% LCM (*p* = 0.017, Figures 3A,B). Similarly, the increase in Eomes expression upon *ex vivo* culture of CD56^bright^ NK cells was significantly reduced by treatment with LCM in a dose dependent manner. By day 7, Eomes MFI in untreated NK cells had risen to 3,149 ± 389.9 from 1,343 ± 204.4 at day 0. Treatment with 5% LCM reduced the MFI to 1,774 ± 630.6, with a further reduction to 983.3 ± 243.1 MFI with 10% LCM (*p* = 0.0278, Figure 3C). At day 7, CXCR6 was expressed on 45.5 ± 6.4% of CD56^bright^ NK cells. This level increased to 58.0 ± 15.5% and 65.3 ± 10.8% with 5 and 10% LCM, respectively (*p* = 0.21, Figure 3D).

Hepatic CD56^dim^ NK cells behave in a similar manner when treated with LCM although the effect is not as pronounced as in CD56^bright^ NK cells. Tbet expression is reduced in a dose dependent manner (untreated MFI 789.7 ± 171.8, 5% LCM MFI 720.7 ± 19.7, 10% LCM MFI 553.2 ± 7.8, *p* = 0.18, Figure 3E). A dose dependent decrease in Eomes expression was also seen, returning the MFI of Eomes to levels seen at day 0 (untreated MFI 2323 ± 4336, 5% LCM MFI 1157 ± 164.9, 10% LCM MFI 736 ± 118, *p* = 0.0331, Figure 3F). No significant change was seen in expression of CXCR6 on CD56^dim^ NK cells (untreated 6.6 ± 2.4%, 5% LCM 3.6 ± 2.1%, 10% LCM 4.1 ± 2.6, *p* = 0.72, Figure 3G).

### Treatment of Peripheral Blood NK Cells With LCM Suppresses Tbet and Eomes Expression but Fails to Induce CXCR6 Expression

**Figure 4:**
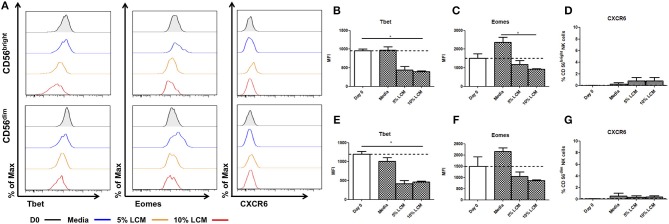
Liver conditioned media suppresses Tbet expression in blood NK cells. NK cells isolated from peripheral blood (PB) were cultured for 7 days, supplemented with 5 or 10% v/v liver conditioned media (LCM) and stained with monoclonal antibodies to assess transcription factor expression. **(A)** Representative histograms of Tbet, Eomes and CXCR6 expression from cells at day 0 (black line), day 7 untreated (blue line), day 7 5% LCM (orange line), and day 7 10% LCM (red line). **(B)** MFI of Tbet in CD56^bright^ NK cells from PB at day 0 and 7 with LCM treatments. **(C)** MFI of Eomes in CD56^bright^ NK cells from PB at day 0 and 7 with LCM treatments. **(D)** Percentage of CXCR6 positive CD56^bright^ NK cells from PB at day 0 and 7 with LCM treatments. **(E)** MFI of Tbet in CD56^dim^ NK cells from PB at day 0 and 7 with LCM treatments. **(F)** MFI of Eomes in CD56^dim^ NK cells from PB at day 0 and 7 with LCM treatments. **(G)** Percentage of CXCR6 positive CD56^dim^ NK cells from PB at day 0 and 7 with LCM treatments. Data presented as mean ± SEM. Data was analyzed using Friedman test, with Dunn's multiple comparison test (*n* = 5; *p < 0.05).

In contrast to hepatic NK cells, blood CD56^bright^ NK cells do not significantly increase their expression of Tbet in culture (Day 0 954.7 ± 47.5 MFI, Day 7 973.5 ± 88.5 MFI, Figures 4A,B). However, treatment with LCM induces reduced Tbet expression after 7 days of treatment (Day 0 954.7 ± 47.5 MFI, Day 7 5% LCM 443 ± 91.2, 10% LCM 395.3 ± 25.2 MFI, *p* = 0.033, Figure 4B). At day 0 the MFI of Eomes was 1,514 ± 237.1 for CD56^bright^ cells, by day 7 the MFI had increased to 2,371 ± 267 (Figure 4C). This was reduced by addition of LCM to 1,177 ± 209 MFI (5% LCM) and 927.7 ± 34.2 MFI (10% LCM). Peripheral blood CD56^bright^ NK cells do not basally express CXCR6 and the receptor was not upregulated at any point during the experiment (Figure 4D).

CD56^dim^ NK cells also undergo a significant decrease in Tbet expression after LCM treatment (day 0 1198 ± 72.8 MFI, Day 7 untreated 1,012 ± 99.1 MFI, 5% LCM 418 ± 83.5 MFI, 10% LCM 464.7 ± 21.5 MFI, *p* = 0.017, Figure 4E). At day 0 the MFI of Eomes was 1,500 ± 420.5 for CD56^dim^ NK cells. At day 7 the MFI had increased to 2,155 ± 163.5, this was reduced to basal levels with the addition of LCM (5% LCM 1,040 ± 199.5 MFI, 10% LCM 859.9 ± 41 MFI, Figure 4F). Expression of CXCR6 on peripheral blood CD56^dim^ NK cells was negligible at all time points (Figure 4G).

### TGF-β, Which Is Present in the Liver Microenvironment, Is Capable of Suppressing Tbet *ex vivo* in Liver Resident and Blood NK Cells

**Figure 5:**
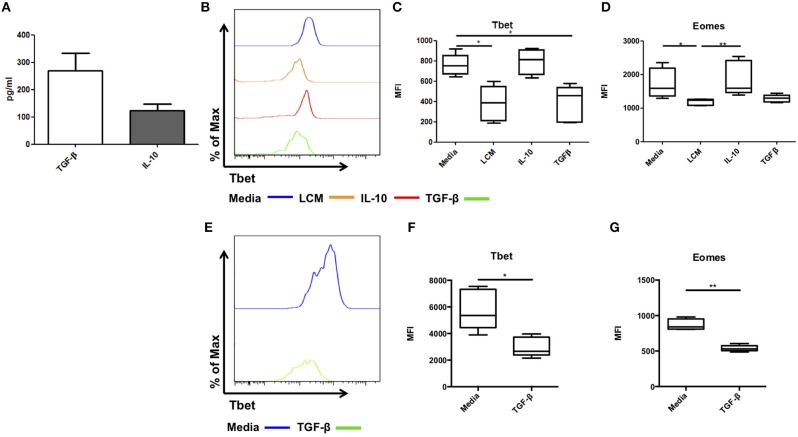
TGF-β can regulate Tbet expression in liver resident and blood NK cells. Liver conditioned media (LCM) was generated from healthy donor liver biopsies. ELISA was performed for the cytokines TGF-β and IL-10. **(A)** Concentration of cytokines TGF-β and IL-10 from matched LCM samples (*n* = 10). CD56^bright^ Eomes^hi^ Tbet^lo^ NK cells FACS isolated from liver perfusate (LP) were cultured for 7 days, supplemented with 10% v/v liver conditioned media (LCM), IL-10 (10 ng/ml) or TGF-β (5 ng/ml) and acquired on a FACS Fortessa. **(B)** Representative histogram of Tbet expression from cells at day 7 untreated (blue line), 10% LCM (orange line), IL-10 (red line), and TGF-β (green line). **(C)** MFI of Tbet in CD56^bright^ NK cells from LP at day 7 with LCM, IL-10 or TGF-β treatments. **(D)** MFI of Eomes in CD56^bright^ NK cells from LP at day 7 with LCM, IL-10 or TGF-β treatments. **(E)** Representative histogram of Tbet expression from blood NK cells at day 7 untreated (blue line) or TGF-β (green line). **(F)** MFI of Tbet in PB CD56^bright^ NK cells at day 7 with or without TGF-β. **(G)** MFI of Eomes in PB CD56^bright^ NK cells at day 7 with or without TGF-β. Data presented as mean ± SEM **(A)** or box and whisker plots with minimum and maximum values **(C,D,F,G)**. Data was analyzed using Friedman test, with Dunn's multiple comparison test or paired *t*-test (*n* = 5; *p < 0.05 and **p < 0.01).

We next attempted to identify the factor present in LCM responsible for maintaining this liver resident phenotype. IL-10 and TFG-β are two immunosuppressive cytokines which can suppress IFN-γ production in NK cells and T cells (14–16). Our group has previously reported the presence of several NK related cytokines, including the essential IL-15, activatory IL-12 and IL-18, and regulatory IL-10 and TGF-β in healthy liver tissue (9). Secreted TGF-β and IL-10 were detected in all LCM samples at concentrations of 270.3 ± 63.1 pg/ml and 124.3 ± 22.3 pg/ml, respectively (Figure 5A). Next, we assessed the effect of IL-10 and TGF-β on the phenotype on hepatic NK cells in culture.

Hepatic CD56^bright^Eomes^hi^Tbet^lo^ NK cells were isolated by FACS sorting. They were cultured in RPMI supplemented with 10% human AB serum and IL-15 (2 ng/ml) for 7 days with or without LCM (10% v/v), IL-10 (10 ng/ml), or TGF-β (5 ng/ml). Media was replenished ever 2–3 days. As before Tbet and Eomes expression increased during culture and LCM significantly reduced the expression of Tbet and Eomes (Figures 5B–D). IL-10 had no effect on Tbet expression (MFI 792.4 ± 56.4, Figure 5C), however TGF-β significantly reduced the expression of Tbet compared to untreated cells (untreated MFI 760.5 ± 46.9, LCM 381.1 ± 78.2, TGF-β 385.2 ± 79.8, *p* = 0.0014, Figure 5C). IL-10 did not significantly alter the expression of Eomes, while TGF-β reduced Eomes expression to a similar level to LCM, however this did not reach statistical significance (Figure 5D).

Treatment of magnetic bead-purified peripheral blood NK cells with TGF-β for 7 days likewise resulted in a significant decrease in Tbet expression in CD56^bright^ NK cells (Day 7 untreated 5,776 ± 679 MFI, TGF-β treated 2,979 ± 328 MFI, *p* = 0.0421, Figures 5E,F). A significant decrease in Eomes expression upon TGF-β treatment was also observed (Day 7 untreated 873 ± 34 MFI, TGF-β treated 539 ± 19 MFI, *p* = 0.0024, Figure 5G).

### Inhibiting TGF-β Signaling Partially Reverses the Effect of LCM Treatment on Liver Resident and Blood NK Cells

**Figure 6:**
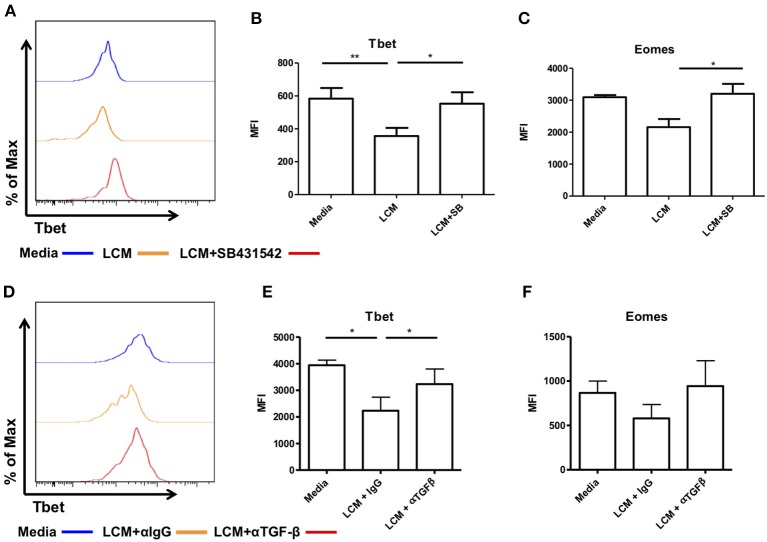
Blocking TGF-β inhibits the ability of liver conditioned media to suppress Tbet and Eomes expression. CD56^bright^ Eomes^hi^ Tbet^lo^ NK cells FACS isolated from liver perfusate (LP) were cultured for 7 days, supplemented with 10% v/v liver conditioned media (LCM) with and without SMAD inhibitor SB431542 and acquired on a FACS Fortessa. **(A)** Representative histogram of Tbet expression from cells at day 7 untreated (blue line), 10% LCM (orange line) or LCM & SB431542 (red line). **(B)** MFI of Tbet in CD56^bright^ NK cells from LP at day 7 with LCM or LCM & SB431542. **(C)** MFI of Eomes in CD56^bright^ NK cells from LP at day 7 with LCM or LCM & SB431542. NK cells isolated from peripheral blood (PB) were cultured for 7 days, supplemented with 10% v/v LCM with 5 μg/mL anti-TGF-β1 blocking antibody or IgG isotype control and acquired on a FACS Canto II. **(D)** Representative histogram of Tbet expression in CD56^bright^ NK cells from peripheral blood at day 7 with LCM or LCM & TGF-β1 blocking antibody. **(E)** MFI of Tbet in CD56^bright^ NK cells from peripheral blood at day 7 with LCM or LCM & TGF-β1 blocking antibody. **(F)** MFI of Eomes in CD56^bright^ NK cells from peripheral blood at day 7 with LCM or LCM & TGF-β1 blocking antibody. Data presented as mean ± SEM. Data was analyzed using Friedman test, with Dunn's multiple comparison test or repeated measures one-way ANOVA (*n* = 3–8, *p < 0.05 and **p < 0.01).

We next inhibited TGF-β receptor signaling using the inhibitor SB431542, which inhibits the TGF-β type I receptor activin receptor-like kinase (ALK)5 and related genes ALK4 and ALK7. Liver CD56^bright^ NK cells were pre-treated with SB431542 for 2 h before being treated with LCM. As before, treatment with LCM significantly reduced the expression of Tbet compared to untreated cells (Figures 6A,B). Pre-treatment with SB431542 prevented the reduction in Tbet expression (LCM: MFI 356 ± 49.6, LCM+SB431542: MFI 552.8 ± 69.6, *p* = 0.0239, Figure 6B). The addition of SB431542 also significantly increased the expression of Eomes compared to LCM treated cells (Figure 6C).

In order to confirm that this effect was specific to TGF-β receptor signaling we next treated magnetic bead-purified blood NK cells with LCM and 5 μg/ml of a TGF-β1 blocking antibody (clone 19D8, BioLegend) or isotype control (clone MOPC-21, BioLegend). Blocking TGF-β1 prevented the inhibition of Tbet in CD56^bright^ NK cells (Figures 6D,E). Blocking TGF-β1 also prevented the inhibition of Eomes by LCM although this did not reach statistical significance (Figure 6F).

## Discussion

In this study, we have shown that the liver microenvironment is essential for maintaining the unique Eomes^hi^Tbet^lo^ phenotype of liver resident NK cells. NK cells isolated from liver perfusate lose their unique Eomes^hi^Tbet^lo^ phenotype when cultured, but this can be reversed by the addition of LCM. The liver is rich in immunoregulatory cytokines, including TGF-β and IL-10, produced by Kupffer cells and immature dendritic cells (9, 17–19). Liver resident NK cells cultured with TGF-β, but not IL-10, maintain their Eomes^hi^Tbet^lo^ phenotype, indicating that TGF–β is essential for maintaining liver resident populations. Blocking TGF-β signaling through pre-treatment with SB431542 reverses this effect and shifts the phenotype of liver resident NK cells to a peripheral blood-like state. Furthermore, Tbet expression in peripheral blood NK cells can be modulated by treatment with both LCM and TGF–β.

Culture with LCM can maintain CXCR6 expression, but it does not appear to induce expression of CXCR6 on peripheral blood NK cells. Cytokine stimulation (IL-12, IL-15) of peripheral blood NK cells can induce CXCR6 or CD49a expression, however the level of CXCR6 expression was highly variable and less than half of the stimulated NK cells showed expression, suggesting additional stimuli are required for sustained CXCR6 expression (20). NK cells may acquire CXCR6 expression in the periphery, through cytokine activation, and migrate to the liver. Here CXCL16, RANTES, and CCL3, present in the liver sinusoid, may retain activated NK cells (4, 21) and possibly sustain CXCR6 expression. Following recruitment to the liver sinusoid, TGF-β (produced by resident macrophages and dendritic cells) may alter transcription factor expression and repress the production of pro-inflammatory cytokines (IFN-γ).

Significant work has been performed investigating the effect of TGF-β on NK cells. TGF-β has been shown to have potent immunosuppressive effects and alters NK cell development (15, 22). Furthermore, TGF-β has been shown to suppress glycolysis and inhibit the pro-inflammatory effector functions of CD56^bright^ NK cells, such as IFN–γ production (23). The mechanism by which TGF-β inhibits Tbet and IFN-γ has previously been elucidated in T cells, where TGF-β signaling induces the expression of SHP-1 which in turn inhibits STAT1/4 and Tbet expression (24, 25). Recently, TGF-β has been shown to be involved in the conversion of NK cells to ILC1-like cells, via a JNK-dependent, Smad4-independent pathway resulting in reduced cytotoxicity in murine tumor models (26, 27). Why this process does not appear to occur in liver resident NK cells warrants further investigation. Intriguingly, Smad4 appears to suppress the JNK-dependent non-canonical TGF-β signaling associated with differentiation into ILC1-like cells within tissues, indicating that canonical TGF-β signals may be required to maintain NK cell phenotype. It is important to note there is a marked differences in the phenotype and function of liver resident NK cells between humans and mice (28). Furthermore, single cell RNA-seq analysis of human and murine NK cells have highlighted similarities but also significant differences in peripheral blood and splenic samples (29). Therefore, TGF-β may have divergent functions between species and care must be taken when comparing murine and human data.

In this study, we identified TGF-β as a factor required for the induction and maintenance of the tissue resident phenotype of liver resident NK cells in humans. Liver resident NK cells are unique in that they can provide immunosurveillance without producing large quantities of IFN-γ with its potential to drive tissue damaging pathology. In this context, liver resident NK cells can remove dysplastic or virally infected cells without perturbing the tolerogenic milieu of the liver, which so often leads to chronic inflammatory conditions. Further evidence for the importance of TGF-β in the establishment and maintenance of tissue resident NK cell populations in humans comes from analysis of uterine NK cells. Uterine NK cells share many phenotypic characteristics with liver resident NK cells (CD56^bright^Eomes^hi^CD69^+^) (30). The local uterine microenvironment is rich in TGF-β and treatment of blood NK cells with either endometrial stromal cell supernatants or TGF-β alone can induce phenotypic changes resembling decidual NK cells (31).

Our results suggest that disruption of TGF–β levels in the liver microenvironment in disease could lead to a loss of local immune regulation and the promotion of tissue inflammation and damage. While the roles of liver resident NK cells in maintaining homeostasis or mediating liver disease have yet to be established in humans, a reduction in TGF-β levels during liver disease could drive resident NK cell populations to a more conventional pro-inflammatory phenotype and contribute to chronic inflammation. In this situation, restoring homeostatic cytokine levels would be essential to regulate local immunity and tissue repair in the liver. Interestingly, the use of a strong pro-inflammatory signal, such as PMA, appears to overcome this TGF-β mediated repression and restore IFN-γ production (2, 5). This appears similar to the mechanism by which TGF-β imprinted peripheral blood NK cells become hyper-producers of IFN-γ *in vitro* (32). While in healthy liver these resident NK cells have suppressed pro-inflammatory function, it appears this can be overcome with sufficient stimulation.

The liver is a naturally tolerogenic environment, which maintains unique anti-inflammatory status even in the presence of immune activating dietary antigens and bacterial components. It is therefore no surprise to find that liver resident NK cells are profoundly changed by residing in this microenvironment. TGF-β is one of the chief mediators of the tolerogenic hepatic environment and we have shown here that this regulation extends to the phenotype and function of NK cells. Through the suppression of Tbet, liver resident NK cells have reduced pro-inflammatory potential but maintain their ability to perform immunosurveillance in an organ prone to infection and malignancy.

## Ethics Statement

All protocols were approved by St. Vincent's University Hospital Ethics Committee and the Trinity College Dublin School of Medicine Research Ethics Committee, in accordance with the ethical guidelines of the 1975 Declaration of Helsinki. Patients were consented pre-operatively by DH, EH, or JG. All patients were supplied with a information packet informing them of the project aims, their rights to withdraw at any time and the length of time samples and data would be retained. All patients were over the age of 18 and had no conditions which would preclude them from giving informed consent.

## Author Contributions

CO, MR, and CH contributed to the conception and design of the study. DH, EH, and JG acquired data and managed clinical samples. CH, GJ, and DA designed and carried out experimental procedures. Statistical analysis was performed by CH, GJ, and MR. CO, MR, CH, DH, EH, and JG were involved in interpretation of data and manuscript preparation. Manuscript drafting was performed by CH, MR, and CO. All authors reviewed and approved the final manuscript.

### Conflict of Interest Statement

The authors declare that the research was conducted in the absence of any commercial or financial relationships that could be construed as a potential conflict of interest.
